# Glutathione Supplementation in Parenteral Nutrition to Prevent Oxidative Stress Related Complications in Preterm Infants: A Narrative Review of Preclinical Efficacy and Safety Profile

**DOI:** 10.3390/nu18132092

**Published:** 2026-06-26

**Authors:** Jean-Claude Lavoie, Ibrahim Mohamed

**Affiliations:** 1 Department of Nutrition, CHU Sainte-Justine, Université de Montréal, Montreal, QC H3T 1C5, Canada; jean-claude.lavoie@umontreal.ca; 2 Department of Pediatrics-Neonatology, CHU Sainte-Justine, Université de Montréal, Montreal, QC H3T 1C5, Canada

**Keywords:** prematurity, neonatal intensive care unit, parenteral nutrition, photoprotection of parenteral nutrition, glutathione, intravenous GSSG, oxidative stress, peroxide, toxicity, adverse effect

## Abstract

Numerous adverse effects caused by oxidative stress are commonly observed in preterm infants. This stress is caused by the oxidative burden resulting mainly from supplemental oxygen and parenteral nutrition (PN), and by their precarious antioxidant defense. The natural antioxidant defense against these oxidant molecules relies on glutathione, levels of which are low in preterm infants. Given that several short- and long-term biological complications are associated with this oxidative stress, the aim of this narrative review was to discuss glutathione supplementation as a method for reducing it. Consequently, after briefly discussing the effectiveness of partially reducing oxidative stress through adequate photoprotection of PN, it is proposed to correct premature infants’ glutathione deficiency by adding glutathione to PN. To justify the proposed intervention, this article will discuss the scientific evidence related to the (1) importance of glutathione, (2) causes of glutathione deficiency and ways to prevent it, (3) reasons why the disulfide form (GSSG) is recommended over the reduced form (GSH) for PN supplementation, (4) efficacy of parenteral GSSG in preventing oxidative stress, and (5) safety profile of glutathione infusion. In conclusion, we believe that the time has come to improve the health of premature infants by providing GSSG-supplemented PN.

## 1. Introduction

Due to the immaturity of their lungs and gastrointestinal system, premature newborns, especially those born before 30 weeks of gestation, require supplemental oxygen and parenteral nutrition (PN). Even though these treatments are essential for their life and development, they were found to be the main sources of oxidative stress in this population [1], and they are associated with severe morbidity such as bronchopulmonary dysplasia (BPD), retinopathy of prematurity (ROP), necrotizing enterocolitis (NEC), etc. Multiple reviews published during the last 20 years highlight the important role played by oxidative stress in these clinical complications in premature infants [2,3,4], hence the importance of reducing exposure to oxidants and/or improving the antioxidant defenses of newborns.

Oxygen is the most powerful electron acceptor in biology. When the fraction of inspired oxygen (FiO_2_) is 0.21 in the lungs, these oxygen molecules bind to the hemoglobin in red blood cells for transport. When FiO_2_ exceeds 0.21, hemoglobin saturation may be exceeded, allowing “free” O_2_ molecules to capture electrons from its surroundings (double bonds in fatty acids, proteins, or vitamin C, etc.) to form the superoxide anion (^•^O_2_^−^) that is rapidly converted into hydrogen peroxide (H_2_O_2_) by superoxide dismutases. H_2_O_2_ is an important oxidizing agent in biology. An FiO_2_ ≥ 0.25 is sufficient to observe oxidation of the blood redox potential of glutathione in preterm infants [5,6]. Advances in neonatal care have led to a significant reduction in the level of oxygen supplements through continuous monitoring of the premature infant’s blood saturation level. Today, the level of oxygen supply cannot be reduced any further without serious consequences, including increased mortality, for these newborns [7].

A second important source of oxidative stress in this population is PN. These nutritive solutions carry heavy loads of oxidizing molecules such as peroxides [4]. In order to reduce the oxidant load of PN and the associated medical complications observed in neonatal intensive care units (NICUs), several scientists are calling for a new formulation to optimize PN. The authors share the same goal of improving the health of premature infants by optimizing their PN. Two approaches are possible to reduce oxidative stress in this population: reduce the oxidant load exposure and/or improve the capacity of newborns to detoxify these oxidant molecules received through their PN and oxygen supplement.

Spontaneously generated peroxides in PN are responsible for the oxidative stress observed in animals and humans receiving PN [4]. A PN solution containing 1% MVP generates approximately 300 µM of peroxides within three hours of mixing. If the solution is fully photoprotected, the peroxide concentration is halved to around 150 µM [4,8]. The discovery that peroxide levels can be modulated simply by photoprotecting PN has made it possible to evaluate the biochemical and biological effects of these peroxides. Studies conducted in vitro and in vivo on newborn guinea pigs are conclusive, as noted by Lavoie JC and Chessex P in a review published in 2019 [4]. Newborn guinea pigs infused during 4 days with PN containing 1% MVP or infused with different peroxides generated in PN at concentrations measured in PN resulted in lower levels of glutathione in the lungs, high oxidative stress (oxidized redox potential), high procollagen mRNA, high apoptosis, and hypo-alveolarization. All these effects were avoided with PN photoprotection. Thus, in animals, a concentration of 300 µM peroxides is toxic, while 150 µM (in photoprotected PN) seemed safe. In humans, two single-center RCTs were conducted, of which one was a post hoc analysis of data [9] from a previous study testing the impact of photoprotection on advancement of minimal enteral nutrition, to compare the incidence of BPD in premature infants receiving PN containing 1% MVP in a photoprotected versus control group reported, in 2007 and 2009, a lower incidence of BPD in newborns receiving photoprotected PN [9,10]. In 2015, the multicenter RCT conducted by Laborie S et al. [11] failed to demonstrate a significant impact on the incidence of BPD; it should be noted that the level of MVP in PN was heterogeneous according to individual hospital practice. A meta-analysis bringing together four RCTs, including the three cited above, demonstrated in 2017 [12] a 50% reduction in mortality in premature infants benefiting from PN photoprotection.

While photoprotection of PN is a key intervention that reduces oxidative stress in preterm infants, its effectiveness could be limited. We must therefore help the newborn develop its antioxidant capacity. Oxidizing molecules to which newborns on PN are exposed are mainly peroxides. The natural antioxidant system against these molecules is glutathione, levels of which are low in premature infants [2,13,14,15,16]. Our team then proposed and tested the effects of adding glutathione to PN [17]. Given the growing body of preclinical evidence demonstrating that it is possible to improve the newborn’s ability to detoxify these molecules by adding glutathione to their PN, this narrative review aims to discuss the biochemical mechanisms underlying this concept and to review scientific literature reporting its efficacy and safety.

## 2. Methods

As glutathione deficiency in preterm infants was confirmed in the 1990s and continued to be observed in the recent literature [2,13,14,15,16], we identified scientific works concerning this deficiency, exploring its causes, describing the efficacy and safety profile starting from 1990 until the present. The following combinations of keywords were used in the PubMed search engine: parenteral nutrition; light protection; toxicity; prematurity; glutathione; intravenous glutathione. Relevant articles published between 1990 and 2026 were selected to provide the data presented in the tables.

## 3. Results

### 3.1. Importance of Glutathione

The unwanted (toxic level) molecules, such as peroxides and even aldehydes resulting from lipid peroxidation generated in PN or as a result of oxygen exposure, are detoxified by an efficient system involving glutathione peroxidases and glutathione S-transferases. However, the co-substrate glutathione is low in premature infants [2,13,14,15,16]. In plasma, glutathione concentration (1.2 ± 0.1 µM) [16] represents approximately 25% of the normal value [18,19,20]. The consequence of this low glutathione level is the low detoxification activity by these enzymes. Thus, the impacts of the intracellular concentration of peroxides are closely linked to glutathione metabolism.

Intracellular concentrations of H_2_O_2_ act as a powerful modulator of several metabolic processes by oxidizing cysteine residues present in various enzymes, thereby altering their activities. The oxidized thiol group (sulfenic form, -SOH) reverts to its reduced form (thiol form, -SH) depending on the cell’s redox potential, which is primarily controlled by the GSSG:2GSH couple. A high (oxidized) redox potential favors the maintenance of the sulfenic form, while a low (reduced) redox potential allows reduction to the thiol form [21]. Furthermore, the Nernst equation, which quantifies the redox potential, indicates that low glutathione levels, as well as high GSSG levels (the oxidized form of glutathione), correspond to an oxidized redox potential, and thus to oxidative stress [22].

The cellular redox value is a regulator of several cell functions. For example, the dependence of the cell cycle on the redox value present in the cell was reported; indeed, when a cell transitions from a reduced to an oxidized redox value, the cell will evolve from proliferation to differentiation, and then to apoptosis [22]. Therefore, the maintenance of homeostasis in the intracellular variation in the redox potential over time enables organogenesis. This is particularly important in premature newborns, whose many organs are still developing. As mentioned above, in newborn guinea pigs, the PN-induced oxidation of redox potential is associated with exaggerated apoptotic events and loss of alveoli [4,17,23,24], while in human premature neonates, the redox potential value measured in blood at one week of life is associated with the severity of BPD [5].

Adequate photoprotection remains a challenge for the neonatal intensive care units (NICUs) [25]. Even with the new proposition in the literature [26], photoprotection reduces the amount of peroxide generated by half. Thus, there is still a need for improving the antioxidant capacity of premature newborns by correcting their glutathione deficiency to be able to detoxify the remaining peroxides even after effective photoprotection of PN is applied [4,24,27,28].

### 3.2. What Is the Origin of This Glutathione Deficiency in Preterm Infants?

As the enzymatic synthesis of glutathione is mature in premature newborns [29], a lack of substrate has been suspected and demonstrated. Glutathione is a tripeptide (γ-glutamylcysteinylglycine, GSH) whose synthesis is limited by the cellular availability of cysteine [30,31]. In adults, cysteine is not an essential amino acid because it can be endogenously synthesized from methionine through the transmethylation and the transsulfuration pathways. However, in premature newborns, cysteine is considered a conditionally essential amino acid since the last enzyme in this cascade, γ-cystathionase, is immature [32,33] while the activity of the first enzyme, methionine adenosyltransferase, is reduced by the peroxides contaminating PN, as demonstrated in animals [34,35]. This low transformation of methionine to cysteine leads to cysteine deficiency. Cysteine deficiency is well known in premature babies [27,36,37,38].

In a situation of low cysteine availability, protein synthesis is favored compared to that of glutathione. Indeed, cysteinyl-tRNA synthetase has a greater affinity for cysteine (Km = 1–20 μM [39]) than the first enzyme in GSH synthesis, γ-glutamylcysteine ligase (Km = 200–350 μM [40,41]). Cells must therefore contain sufficient cysteine to maintain both protein and glutathione synthesis. Otherwise, a low glutathione concentration can occur.

### 3.3. Possible Sources of Cysteine to Sustain Glutathione Synthesis in Parenterally Fed Infants

Although some parenteral amino acid preparations contain cysteine or an addition of the hydrochloride form of cysteine, cellular uptake of cysteine is immature in preterm neonates [42]. Another possibility is to add N-acetylcysteine, which is a pro-cysteine that is more soluble than cysteine, but it has limited bioavailability. In addition, N-acetylcysteine was suspected to be poorly deacetylated in premature infants [43]. A Cochrane meta-analysis reported that the addition of cysteine or N-acetylcysteine to PN failed to improve the glutathione concentration in premature infants or reduce the incidence of BPD [44].

The natural source of cysteine for cells is glutathione itself. Due to its abundance (1–10 mM in cells) and high cysteine content, glutathione is considered a cysteine reservoir for the organism [45,46,47]. Through the γ-glutamyl cycle, cysteine from plasma glutathione is transferred into the cell [48,49]. The ectoenzyme γ-glutamyl transpeptidase (γGT) present at the surface of cells hydrolyzes the gamma-peptide bond of extracellular glutathione, releasing the dipeptide cysteinylglycine, which is captured by the cell [50,51], where dipeptidases release the cysteine into the cytosol [52]. Therefore, plasma glutathione (both GSH and GSSG) can be considered a universal pro-cysteine molecule for different cells. The natural substrates of γGT are molecules containing a γ-glutamyl group, such as reduced glutathione (GSH), disulfide glutathione (GSSG), and glutathione-conjugated molecules [49,53,54]. The affinity of the enzyme (Km) for these forms of glutathione varies between 6 and 12 µM [53], whereas the normal concentration of glutathione in plasma is reported to be between 3 and 12 µM [18,19,20]. The similar values between Km and normal glutathione concentration highlight the importance of the latter on enzyme activity and its effectiveness in supplying cysteine to cells. Unfortunately, the plasma concentration of glutathione in 1-week-old premature infants is 1.2 ± 0.1 μM [16]. The metabolic defect leading to this glutathione deficiency originates in the liver. Indeed, plasma glutathione mainly comes from the liver, where it is actively exported by a sinusoidal transporter [55]; GSH does not freely cross cell membranes. The high rate of glutathione synthesis by this organ is explained by the high availability of cysteine following the transmethylation and transsulfuration of methionine. In premature neonates, however, as mentioned above, the metabolic efficiency of this pathway is reduced by the immaturity of γ-cystathionase and the inhibitory action of PN peroxides on methionine adenosyltransferase. Newborn animals fed exclusively with PN had low plasma and lung glutathione levels [21], which were prevented by the addition of glutathione in their parenteral nutrition [21].

### 3.4. GSSG Rather than GSH for PN Supplementation

γGT, which is mature in the first days of life of premature newborns [29], has a similar affinity for GSH and GSSG [53]. The predictable reactivity of GSH with other components of PN explains its low stability; indeed, after 24 h at room temperature, 11% glutathione remained in GSH-supplemented PN solutions compared to 72% in GSSG-supplemented solutions [21]. The loss of 28% of glutathione is explained by a disulfide exchange between GSSG and cysteine present in parenteral nutrition, generating the mixed disulfide cysteine-glutathione [56]. The latter molecule is also a substrate for γGT, providing cysteine to cells for de novo glutathione synthesis [57]. All our animal studies using GSSG supplementation of PN were successful in preventing PN-induced oxidative stress and biological injuries [17,21,23,24,35,58].

While it may appear less intuitive to administer GSSG to prevent oxidative stress, it should be noted that GSSG supplementation has a very short half-life in the plasma (less than 15 min) and it is rapidly used by γGT to provide the intracellular cysteine necessary for GSH synthesis in all tissues, including the liver, as shown in animals [21], enabling it to release more GSH into the plasma and thereby promoting a return to normal plasma redox potential. This was confirmed in our animal studies directly measuring redox potential in animals receiving PN supplemented with GSSG [21].

### 3.5. Efficacy of GSSG Supplementation

Newborn guinea pigs were used as a neonatal model of PN to address certain questions regarding the biochemical mechanisms involved. It has been demonstrated that PN, or the peroxides generated during PN, induces low levels of plasma glutathione in these animals [21] and, in the lungs, a decrease in glutathione [59], as well as a significant increase in redox potential (a marker of oxidative stress) [17,23,24,60,61], excessive apoptosis, alveolar simplification (the main characteristic of BPD) [17,23,24,60,62], and increased expression of the procollagen gene (a precursor of fibrosis or a tissue remodeling index) [63]. As summarized in Table 1, the addition of GSSG to PN prevented all these manifestations [17,24,63]. The effects of oxidizing molecules present in PN are also observed in the liver. For example, these include the accumulation of lipid droplets or steatosis [58], oxidative stress [21,58], and gene methylation [64]. This last observation pertains more specifically to the metabolism of endogenous H_2_O_2_ and glutathione, which play a role in several metabolic processes, particularly those related to glucose and lipid metabolism [58,65,66]. This hypermethylation persisted for at least fifteen weeks after the discontinuation of parenteral nutrition [64]. All these effects were prevented by enriching PN with GSSG (Table 1). In newborn New Zealand rabbits, Hong L et al. [34] also reported similar protective effects of GSH supplementation (Table 1) on markers of oxidative stress, cholestasis, steatosis, and apoptosis [34]. These findings underscore the importance of correcting glutathione deficiency in preterm infants receiving parenteral nutrition.

### 3.6. Safety Profile of Intravenous Glutathione Administration

A dose–response study in animals [24] demonstrated that lung damage caused by PN infusion could be prevented with a GSSG dose of 0.33 mg/kg/d, while the prevention of plasma glutathione deficit was achieved with a dose of 0.75 mg/kg/d. This suggests that the dose required to achieve a steady-state concentration of glutathione in plasma ensures its effectiveness in preventing lung damage. Thus, in an RCT, we anticipate that correcting plasma glutathione deficiency early in the life of a preterm infant will ensure the prevention of BPD, which is typically diagnosed several weeks after the discontinuation of PN.

In this preclinical study, the plasma total glutathione concentration reached a plateau (5.5 µM) in animals that received a dose between 0.75 mg/kg/d and the maximum dose used (1.3 mg/kg/d). This plateau can be explained by the short half-life of glutathione in plasma, which is approximately 15 min in rats [69] and humans [70], with glutathione being eliminated in large quantities via urine [70]. This animal study suggests a range of GSSG to be used in a clinical trial to correct glutathione deficiency in premature infants receiving PN. It is certainly reasonable to expect a greater need for GSSG in preterm infants. Oxygen therapy and the infant’s growth rate must be taken into account. However, the dose to be used would be of the same order of magnitude. At such a dose, the short half-life would also prevent an accumulation of glutathione in plasma. GSH overaccumulation is also difficult in cells. This is because GSH production is limited by the negative feedback of GSH on its own synthesis [48,71,72]. The inhibition constant (Ki) of GSH on γ-glutamylcysteine ligase, the first enzyme in GSH synthesis, is 0.4 to 0.5 mM, which is close to the intracellular concentration of GSH [72]. Thus, at this dose of GSSG, no harmful effects are expected. However, twenty-four years ago, Stabler SP and colleagues [68] reported a possible side effect in newborn baboons of a 9-day infusion of GSH at 354 mg/kg/d on the arterio-alveolar oxygen gradient. The authors suspected a greater availability of nitric oxide (NO). We know that NO is transported by GSH (binding with the thiol function). By using GSSG instead of GSH, at a much lower dose (300 times less), this interaction with NO is unlikely.

Thus, in a clinical trial aimed at correcting plasma deficiency, it is highly unlikely that such a low dose of GSSG would cause an adverse reaction. We analyzed safety data from animal studies using PN with added glutathione (Table 1) and a mix of human and animal studies testing intravenous GSH administration without PN (Table 2). This safety profile section provides a concise and precise description of the experimental results of the studies included, their interpretation, as well as the conclusions and the reported safety concerns.

In the nine animal studies presented in Table 1, only two possible adverse effects of glutathione infusion were reported. The first is the baboon study mentioned in the previous paragraph, where GSH was added to PN during the first days of life of premature baboons, who thus received 354 mg of GSH/kg/d. The second reported adverse effect is a higher degree of steatosis (lipid droplets in the liver) in the one-month-old guinea pig group receiving PN enriched with 1.1 mg GSSG/kg/d compared to the PN or control groups. This last observation remains without explanation. Another guinea pig study (3-day-olds) reported a lower steatosis level in animals receiving PN supplemented with the same dose of GSSG compared to the PN group [58]. Hong L et al. [34] observed lower steatosis in Newborn New Zealand rabbits that received PN supplemented with 20 mg GSH/kg/d.

Table 2 shows eleven (adult) human and two (adult) animal studies testing intravenous administration of GSH without PN. This heterogeneous group of studies was purposely included in this review to describe the safety profile of intravenous glutathione, regardless of its possible indications, dose, and administration schedule. The purpose of administering intravenous GSH in these human studies varied considerably: oral glucose tolerance, ethanol bolus toxicity, Parkinson’s disease symptoms, oncologic treatment toxicity, peripheral neuropathy severity, oxidative stress in patients with impaired renal function, septic shock, or ST-elevation myocardial infarction (STEMI). Doses ranged from a bolus of 8 to 40 mg/kg infused over 10 min to 2 h, 2 to 6 boluses of 16, 19, or 74 mg/kg/d over 4 to 18 months, 32 mg/kg/d over 3 days, or 70 mg/kg/d continuously over 5 days. The two animal studies (adult rats) received a bolus of 154 mg GSH/kg or an increasing dose from 81 to 246 mg GSH/kg infused in 2 h. In both human and animal studies, no adverse effects have been reported.

## 4. Discussion

Oxidative stress is at the heart of scientific research, as evidenced by the number of publications (over 185,200) since 2020 indexed on PubMed under the term “oxidative stress”. In premature infants on PN, the correlation between oxidative stress and major pathological complications is well known [2,3,4]. However, the biochemical mechanisms underlying the long-term effects of oxidative stress are more difficult to identify in this population. Epigenetic phenomena are suspected. Epigenetic phenomena are increasingly linked to oxidative stress. PubMed has recorded more than 4100 articles since 2020 containing the terms “oxidative stress AND epigenetic.” The fact that only three articles have been indexed in PubMed since 2020, when the term “parenteral nutrition” was added to this search (oxidative stress AND epigenetic), and only two when the term “newborn” was added to those, reflects the emergence of a new field of scientific research and a growing concern about the future of premature infants who rely on parenteral nutrition. Thus, to ensure these infants remain healthy throughout their lives, it is essential, first and foremost, to prevent oxidative stress during the neonatal period. The oxidative stress observed in this population is primarily due to their exposure to oxygen and PN, which is contaminated with peroxides, and the low antioxidant defense caused by glutathione deficiency. This review discusses a proposed intervention to increase the antioxidant capacity of premature infants by administering GSSG, as a form of pro-cysteine, via their PN. The aim of this review was to describe the biochemical mechanisms underlying this intervention and to report the scientific evidence regarding its safety and efficacy. Even though the data from this study support the addition of GSSG to PN, this review has some limitations.

The first of these limitations is our incapacity to determine the amount of peroxides that reaches the cells and organs. In the Introduction section, we mention that a PN solution containing 1% multivitamin preparation generates about 300 µM peroxides, as measured after 3 h incubation. The level of peroxides in PN varies over time. What is measured is the concentration at equilibrium between its generation and its destruction by reactions with other components of PN. Friel J showed [86] that peroxide levels rise for up to 3 h, reach a plateau between 3 and 6 h, and then decline during exposure to low nighttime light, before rising again in the morning with daylight in the neonatal intensive care unit. Thus, the level of peroxides received by infants varies throughout the day of infusion. The next question will be how much of this infused H_2_O_2_ will the organs or specific cells receive? A physiologically plausible scenario is that some of the infused H_2_O_2_ will first be reduced by catalases present in erythrocytes; H_2_O_2_ will easily diffuse through the cell membrane. In addition, because the peroxide concentration in urine rises after the introduction of a multivitamin preparation in PN [4], we can speculate that a part of the infused peroxides is also excreted in the urine. Beyond the challenge of accurately estimating the amount of peroxides infused into which organs and cells are exposed, it is reasonable to believe that the infusion of such a large amount of peroxides (nutritive solutions containing µM range of peroxides) must disrupt the cellular environment where the peroxides’ concentration is in the nM range [87]. In fact, it has been clearly demonstrated that adequate photoprotection of PN, which cuts peroxide production in half, has beneficial effects in premature infants [4].

Another consideration is the impact of molecules other than H_2_O_2_ generated in PN. If the concentration of peroxides decreases during the time of infusion, it is because they react with other molecules present in PN, thereby generating new molecules—possibly as yet unknown—that could prove to be toxic. Other nutrients are oxidized when exposed to light, producing byproducts that may be toxic. For example, the lipid emulsion used in parenteral nutrition contains unsaturated fatty acids that are susceptible to oxidation, leading to the formation of organic peroxides and aldehydes, which are highly reactive molecules [88]. Many of these undesirable molecules are also likely to contribute to oxidative stress in preterm infants. In fact, their detoxification occurs via the glutathione system, either through reduction by glutathione peroxidase (e.g., organic peroxides) or through conjugation with a glutathione group by γ-glutamyl transpeptidases (e.g., aldehydes), in order to facilitate their excretion [88]. In a person with impaired GSH synthesis, for example, due to a lack of substrate, as seen in premature infants, these molecules can only exacerbate oxidative stress by consuming GSH.

Even though the presented evidence argues in favor of adding glutathione to PN, the fact that only one research team has recently developed the use of GSSG as a pro-cysteine molecule for PN can also represent a limitation in the supporting evidence of its efficacy. However, the numerous conclusive studies from this group confirming the proposed metabolic mechanisms provide reassurance regarding the feasibility of the proposed treatment.

Human and animal studies using an infusion of GSH demonstrated that this concept of using glutathione to improve health was tested for a long duration in various conditions, without evolving, to our knowledge, to routine human treatment. Only one study [79] infused GSH continuously over 5 days. The focus of the study, published in 2002, was oxidative stress. The trial concluded that the treatment had beneficial impact in the 10 adults with septic shock, without observations of adverse effects. All other studies listed in Table 2 used a bolus of GSH. With plasma glutathione’s half-life of about 15 min [70], the relevance of these studies may be questionable. However, they can be useful when studying the safety profile of intravenous GSH. The relatively few studies on safety can also be explained by the glutathione metabolism, which suggests an absence or low probability of adverse effects. All our team’s animal studies showed a beneficial impact of continuous infusion of GSSG and demonstrated no significant adverse effects.

If GSSG-enriched PN is administered, should exposure to ambient light and/or the cysteine content of PN be taken into account? In theory, correcting the glutathione deficiency might be sufficient. However, the simultaneous infusion of undesirable molecules generated directly from the PN solution left without photoprotection is not without risk. Both of these approaches should be simultaneously considered.

It is important to remember that photo-oxidation processes lead to the loss of nutrients such as vitamin C [26]. GSSG binds to the cysteine present in PN [56]. This could result in a loss of cysteine. It is already known that cysteine levels are low in preterm infants receiving PN [56]. However, the addition of GSSG as a pro-cysteine is precisely intended to compensate for the cysteine deficiency. Furthermore, the conjugation product, cysteine-glutathione disulfide, is also considered a cysteine donor following the action of plasma γGT [56]. Therefore, at this time, the choice of parenteral amino acid preparation should be determined by the healthcare team, regardless of whether GSSG is supplemented.

To date, the main obstacle to studying GSSG supplementation in PN in preterm infants is the lack of a commercially available GSSG formulation of certified quality that is suitable for human intravenous infusion. The authors hope that this literature review will encourage pharmaceutical companies to start producing a GMP (Good Manufacturing Practice)-quality intravenous formulation of GSSG for available use in humans, particularly for premature newborns on PN. Thus, clinical studies could be undertaken to demonstrate proof-of-concept in this population and, eventually, clinical proof of its benefits.

## 5. Conclusions

This review demonstrates the efficacy and potential benefits of supplementing PN with GSSG. It also establishes the safety profile of glutathione infusion (GSSG and GSH) within PN or without PN. Considering the results of this review, we believe that the time has come to commence clinical studies (phase I–II) to test the hypothesis that supplementing PN with GSSG will decrease oxidative stress and its related complications in preterm infants.

## Figures and Tables

**Table 1:** Studies using glutathione-enriched parenteral nutrition.

Reference (Year)	Species	Concerned Tissues	Days on PN	Intravenous Treatment	Glutathione Dose *	PN Effect	Adverse Effect of iv GSH or GSSG
[34] (2007)	New Zealand rabbit; 1-week-old	Liver (plasma)	10 d	Control (maternal feeding), PN, PN + GSH	20 mg GSH/kg/d	PN caused high direct bilirubin and bile acid; cholestatic or steatotic changes, apoptosis, oxidative stress; all were reduced in PN + GSH.	No mention
[21] (2016)	Guinea pig; 3 d old	Liver	4 d	Control (sham), PN ± 10 µM GSSG	1.1 mg GSSG/kg/d	GSSG prevented PN oxidative stress (redox) by increasing GSH levels.	No mention
[67] (2019)	Guinea pig; 1-month-old	Liver, lung, muscle	5 d	Control, PN ± 10 µM GSSG	1.1 mg GSSG/kg/d	GSSG prevents the PN-induced oxidative stress in the lungs and muscles.	More lipid droplets than with PN.
[58] (2021)	Guinea pig; 3 d old	Liver	4 d, during the first week of life	First week: control, PN ± 6 or 12 µM GSSG; weeks 2–16: mouth feeding	During the first week of life: 0.55 or 1.3 mg GSSG/kg/d	At 1 week, PN increased oxidative stress and perturbation of energy metabolism (activity of GCK, PFK, ACC), and increased lipid droplets (steatosis). All were prevented by adding GSSG in PN. At 16 weeks: induction of reductive stress, prevented by +GSSG; perturbation of energy metabolism, partially prevented by +GSSG	No mention
[64] (2024)	Guinea pig; 3 d old	Liver	4 d, during first week of life	First week: control or PN ± 10 µM GSSG; weeks 2–16: standard food	During the first week of life: 1.1 mg GSSG/kg/d	At 1 week, higher methylation of GPx1 gene; lower gene expression of GPx1, GCLC, GSase; all were prevented in PN + GSSG group. In the PN + GSSG group: lower methylation and higher Nrf2 expression (which is an advantage). At 16 weeks: Same. All suggest a perturbation at short- and long-term glutathione and H_2_O_2_ metabolism in animals without GSSG supplementation.	No mention
[63] (2002)	Guinea pig; 3 d old	Lung	4 d	Dextrose(D), D + H_2_O_2_, D + H_2_O_2_ + 10 µM GSSG, PN ± light exposure	1.1 mg GSSG/kg/d	Increased procollagen mRNA, prevented by GSSG	No mention
[17] (2015)	Guinea pig; 3 d old	Lung	4 d	Sham, PN ± light exposure, PN + 10 µM GSSG peroxide, peroxide + 10 µM GSSG	1.1 mg GSSG/kg/d	PN increased redox potential; increased apoptosis, reduced alveoli number; all were prevented by GSSG.	No mention
[24] (2022)	Guinea pig; 3 d old	Lung	4 d	Increasing dose of GSSG, up to 12 µM.	0; 0.1; 0.24; 0.33; 0.75; 1.3 mg GSSG/kg/d	GSSG has prevented (1) alveolar loss (effective dose: 0.33 mg/kg/d); (2) GSSG has corrected the plasma glutathione deficit (effective dose: 0.75 mg/kg/d); (3) GSSG has reduced oxidative stress (redox potential of glutathione) (effective dose: 0.75 mg/kg/d).	No mention
[68] (2000)	Premature baboons: 125–175 d	Plasma	10 d	PN (no cys) ± cysteine or GSH (0–24 h: 24 µmol/kg/h; after 24 h: 48 µmol/kg/h	First 24 h: 177 mg GSH/kg/d After 24 h: 354 mg GSH/kg/d	Compared with NP, GSH (not cysteine) supplementation increased plasma cysteine and cystathionine concentrations.	Higher A-a oxygen gradient.

PN: parenteral nutrition. * For comparison purposes, all glutathione doses have been converted to mg/kg/d.

**Table 2:** Studies using intravenous administration of GSH without parenteral nutrition.

References (Publication Year)	Species	Objective of the Glutathione Treatment	Control	Dose of GSH	Equivalent Dose in mg/kg *	Time of Exposure	Results	Adverse Effect of GSH Treatment
[73] (1992)	Human (*n* = 6); 72 ± 3 years	Oral or I.V. glucose test	No GSH	1.2 g bolus over 2 h	16 mg/kg	2 h	Improved beta-cell response to glucose	None to report
[74] (1993)	Human (*n* = 10/group); 28–50 years	Damage induced by 40 mL 80% ethanol to the gastric body and antrum	No GSH (placebo)	Bolus (over 10 min) of 2.4 g	32 mg/kg	10 min	Reduced damage	Not reported
[75] (1996)	Human; Parkinson’s disease (*n* = 9/group) average age 66 years	On symptoms	No GSH	Bolus (over 1 h) 600 mg twice a day	2 boluses: 16 mg/kg/d	30 days	Decline in disability	Two patients: fever during the third week, erythema of the skin, irritation, and hardness at injection site
[76] (2009)	Human; Parkinson’s disease (*n* = 10–11/group) adults	On symptoms	No GSH	3 boluses of 1.4 g/week	3 boluses: 18.7 mg/kg	4 weeks	Mild symptoms	“Well tolerated, no safety concerns were identified”
[77] (1997)	Human; ovarian cancer (*n* = 151) mean age: 57 years	Toxicity of cisplatin	No GSH	Bolus of 5.55 g/3 weeks	6 boluses of 74 mg/3 weeks	18 weeks	Decreased depression, emesis, peripheral toxicity, hair loss, shortness of breath	Not reported
[78] (1999)	Human; ovarian cancer (*n* = 40) adults	Toxicity of chemical treatment	No GSH	Repeated bolus of 2.5 g before treatment	Repeated bolus of 33.3 mg/kg before treatment	Bolus of 15 min.	Improved survival; protects against the toxicity of cisplatin	No renal, neuronal, or hearing toxicity
[79] (2000)	Human; septic shock (*n* = 10/group) adults	Oxidative stress markers	No GSH	70 mg/kg/d in continuous	70 mg/kg/d in continuous	5 days	Reduced oxidative indices; improved plasma GSH without change in GSSG	“No adverse effects of GSH administration were observed in the trial”
[80] (2011)	Human; reduced renal function *n* = 7/group adults	Oxidative stress markers (LOOHs in urine) associated with contrast-induced nephropathy (CIN)	No GSH	Infusion 100 mg	40 mg/kg	30 min	2 h after CIN, the elevated levels of markers (LOOH in urine) had completely disappeared	No data
[81] (2018)	Human; (*n* = 3) seniors (authors)	On platelet activation in the presence of PVL-damaged neutrophils	No GSH	Bolus of 600 mg	Bolus of 8 mg/kg	15 min	30 and 60 min after treatment, prevention of activation of platelets in an ex vivo assay	None to report
[82] (2019)	Human; with STEMI *n* = 25/group adults	Oxidative stress markers	No GSH (placebo)	Bolus of 2.5 g	Bolus of 33.3 mg/kg	10 min	After 2 h and 5 d, reduced H_2_O_2_ and isoprostaneF2a production, improved HBA (H_2_O_2_ breakdown activity), and NO availability	“In both groups, no side effects or adverse events during infusion and short observation period were recorded”
[83] (2021)	Human; peripheral neuropathy *n* = 114 in GSH gr and 44 in control) Adults	Incidence and severity of chemotherapy-induced peripheral neuropathy	No GSH	1 bolus of 2.4 g/d on 2 or 3 d before chemotherapy cycle	Bolus of 32 mg/kg/d	2 or 3 d	Reduced peripheral neuropathy	“No change in adverse reaction in multiple myeloma patients. So, no adverse effect of glutathione”
[84] (2004)	Male Lewis rats (250 g) *n* = 8/group	To protect the liver from ischemia–reperfusion damage	Sham and control groups	Continuous infusion for 2 h at rates of 50, 100, 200 µmol/h/kg	61, 123, 246 mg/kg	2 h	Prevent injury	None to report
[85] (2000)	Male Wistar rats (260 g) *n* = 11/group	Plasma homocysteine (which is toxic if too high)	Control (Ringer acetate)	Bolus of 0.5 mmol/kg	Bolus of 154 mg/kg		20 min later, decreased plasma homocysteine	Not reported.

* For comparison purposes, all glutathione doses have been converted to mg/kg/d; if human adult weight was not specified, a weight of 75 kg has been used.

## Data Availability

All data are included in the article.
